# Case Report: A Deletion Variant in the *DCAF17* Gene Underlying Woodhouse-Sakati Syndrome in a Chinese Consanguineous Family

**DOI:** 10.3389/fgene.2021.741323

**Published:** 2021-09-23

**Authors:** Guangmin Chen, Ling Zhou, Qimou Chen, Juan Wang, Peng Jiang, Rufei Shen, Min Long, Houdi Zhou

**Affiliations:** ^1^ Department of Endocrinology, University-Town Hospital of Chongqing Medical University, Chongqing, China; ^2^ Department of Endocrinology, Translational Research Key Laboratory for Diabetes, Xinqiao Hospital, Army Medical University (Third Military Medical University), Chongqing, China

**Keywords:** diabetes mellitus, hypogonadism, alopecia, woodhouse-Sakati syndrome, DCAF17, deletion mutation

## Abstract

Woodhouse-Sakati syndrome (WSS, MIM 241080) is a rare neuroendocrine disease characterized by hair loss, hypogonadism, diabetes, hearing loss, and extrapyramidal syndrome, and is usually caused by mutations in the *DCAF17* gene as an inherited disease. *DCAF17* plays an important role in mammalian gonadal development and infertility. So far, there have been no WSS reports in China. The patient introduced in this case is from a consanguineous family. The main symptoms of the patient were alopecia and gonadal agenesis. Other symptoms such as hearing loss, intellectual disability, and hyperglycemia were remarkable, and these symptoms are often observed in WSS patients. We found a nonsense mutation in the 11th exon of the gene *DCAF17* (Refseq: NM_025000) in the patient and her younger brother, which confirmed the diagnosis of WSS. The genetic results also showed that the mutation was inherited from their healthy first-cousin parents.

## Introduction

Woodhouse-Sakati syndrome (WSS) is a rare neuroendocrine and ectodermal disorder, which is caused by mutations in the gene encoding *DCAF17*. The phenotype of WSS is variable, which is also the reason why this disease is often undiagnosed or misdiagnosed. However, the main symptoms of WSS are alopecia, hypogonadotropic hypogonadism, sensorineural hearing loss, diabetes mellitus, and extrapyramidal movements. This syndrome was first reported by Woodhouse and Sakati (Woodhouse and Sakati, 1983). Currently, fewer than 100 WSS patients have been reported, and most of these patients originated from the Middle East. Some cases have also been recognized in other areas, such as in Europe, Turkey, India, Pakistan, Portugal, France, and Japan. In 2008, *DCAF17*, which is located on chromosome 2q31.1, was discovered as the causal gene for WSS by Woodhouse and Sakati (Sheridan et al., 2015; Hdiji et al., 2016; Louro et al., 2019; Sendur et al., 2019; Shah et al., 2020). According to the HGMD database, so far 24 mutations in *DCAF17* have been reported in the literature, and 18 are responsible for WSS, as listed in Supplementary Table S1. These mutations include three nonsense mutations: c.906G>A, p.(Ser114Term), c387G>A, p.(Trp302Term), and c341C>A, p.(Trp129Term); five intronic mutations that are thought to result in mis-localization of the *DCAF17* gene: c.321+1G>A, c.1422+5G>T, c.1091+2T>C, c.1091+1G>A, and c.1091+6T>G; nine frameshift mutations: c.1238delA, c.270delA, c.270dupA, c.289dupA, c.127-3_127-1delTAGinsAA, c.995delT, c.436delC, c.459-7_499del, and c.50delC; and one start loss mutation (c.1G>A) (Sheridan et al., 2015; Hdiji et al., 2016; Louro et al., 2019; Sendur et al., 2019; Shah et al., 2020). We report the first Chinese case and her younger brother, whose symptoms were suggestive of WSS on clinical presentation. The diagnosis was made by molecular genetic analyses. The proband had a nonsense mutation, and her younger brother also had the same mutation.

## Case Report

**FIGURE 1:**
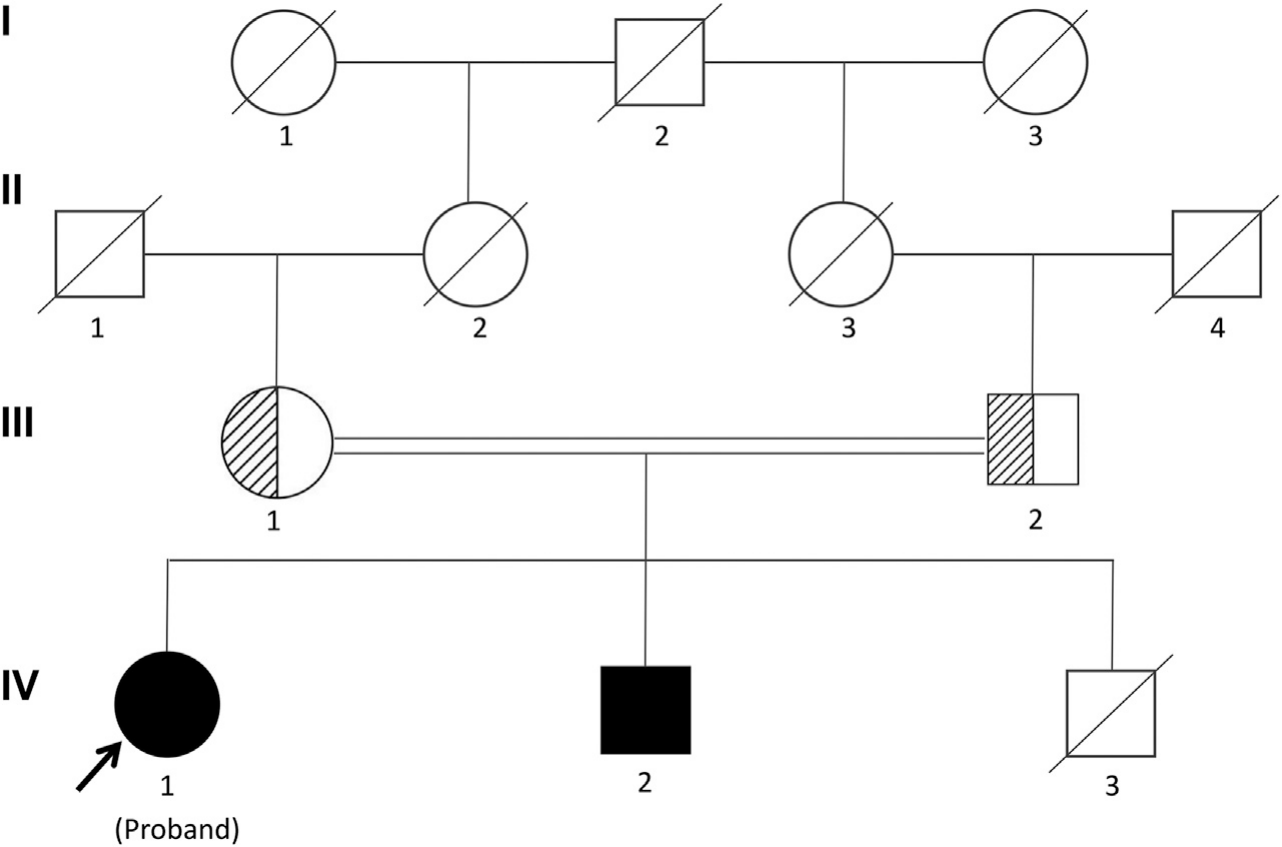
Pedigree of the Chinese family segregating Woodhouse-Sakati syndrome (WSS) in an autosomal recessive manner. Male individuals are represented by squares, and females are represented by circles. Half-filled symbols represent a *DCAF17* heterozygous mutation, while filled symbols represent a *DCAF17* homozygous mutation. The third child of the patient’s parents died at 7 mo and his genetic analyses could not be investigated.

**FIGURE 2:**
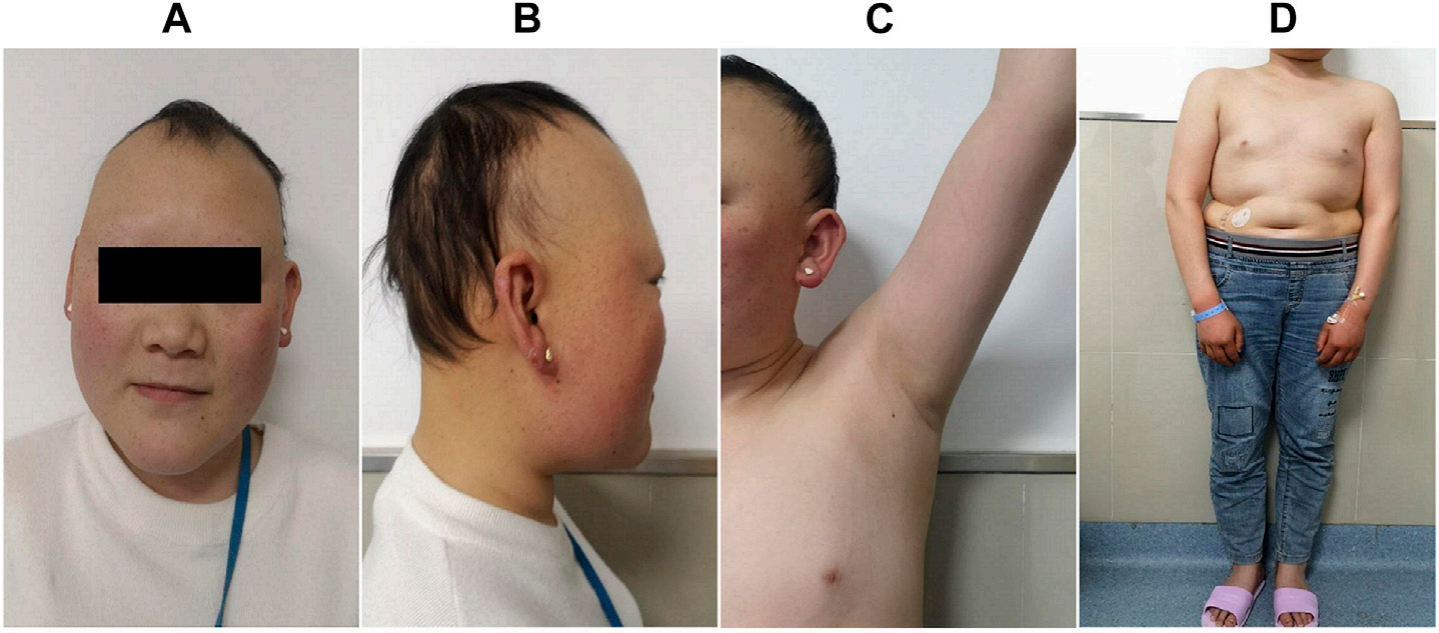
Clinical manifestations of the proband. She presented with sparse hair, especially on both temporal sides and on the forehead, with a progeric face and a large forehead with prominent ears **(A**,**B)**. Secondary sexual characteristics were incompletely developed **(C**,**D)**.

The proband was a 25-yr-old woman, the first child of healthy first cousin parents (Figure 1), with a height of 156 cm and a body weight of 56 kg. She complained of sparse hair and no menstruation. Her mother’s pregnancy, delivery, and feeding history were normal. Her younger brother had similar symptoms, such as alopecia and gonadal agenesis. She had another younger brother died at 7 mo, so his genetic analysis could not be investigated. It is unknown if the cause of death was related to the mutation of this gene. In addition, generations I and II above the patient’s parents died, and genetic analysis could not be performed (Figure 1). During her childhood, her parents noticed that her hair was sparse compared to other children’s. In adolescence, she did not show secondary sexual characteristics. Menarche did not occur. Her previous consultations in other hospitals showed that she had abnormal blood glucose, intellectual disability, dysaudia, an immature uterus and ovaries with few follicles, and her bone age was delayed by 9 yr. Over a year of progynova treatment, she had two menstruations. Then, the patient stopped taking the medicine and no longer menstruated. Physical examination on admission showed that her hair was sparse, especially on both temporal sides and forehead. Her face was progeric, with a large forehead and prominent ears. Secondary sexual characteristics were incompletely developed. Axillary or pubic hair was not present, and minimal breast budding was present with marked vulvar hypoplasia (Figure 2). Unlike some patients in the early literature (Agopiantz et al., 2014), the patient and her younger brother did not have extrapyramidal movement such as dysaudia, dyskinesia with oropharyngeal swallowing disorder, or trouble walking. The patient’s Mini-mental State Examination (MMSE) and Montreal Cognitive Assessment (MoCA) scale scores were 22 and 19 respectively, which confirmed that she had mental retardation. Interestingly, we did not find abnormal extravertebral movement in this patient or her younger brother, which is common in other patients (Agopiantz et al., 2014). The neurological examination showed that the patient had no dysarthria, dysphagia, tremor and abnormal muscle tension; her coordination movements, posture and gait were also normal; At the same time, her muscle strength was normal and Babinski sign was negative.

**FIGURE 3:**
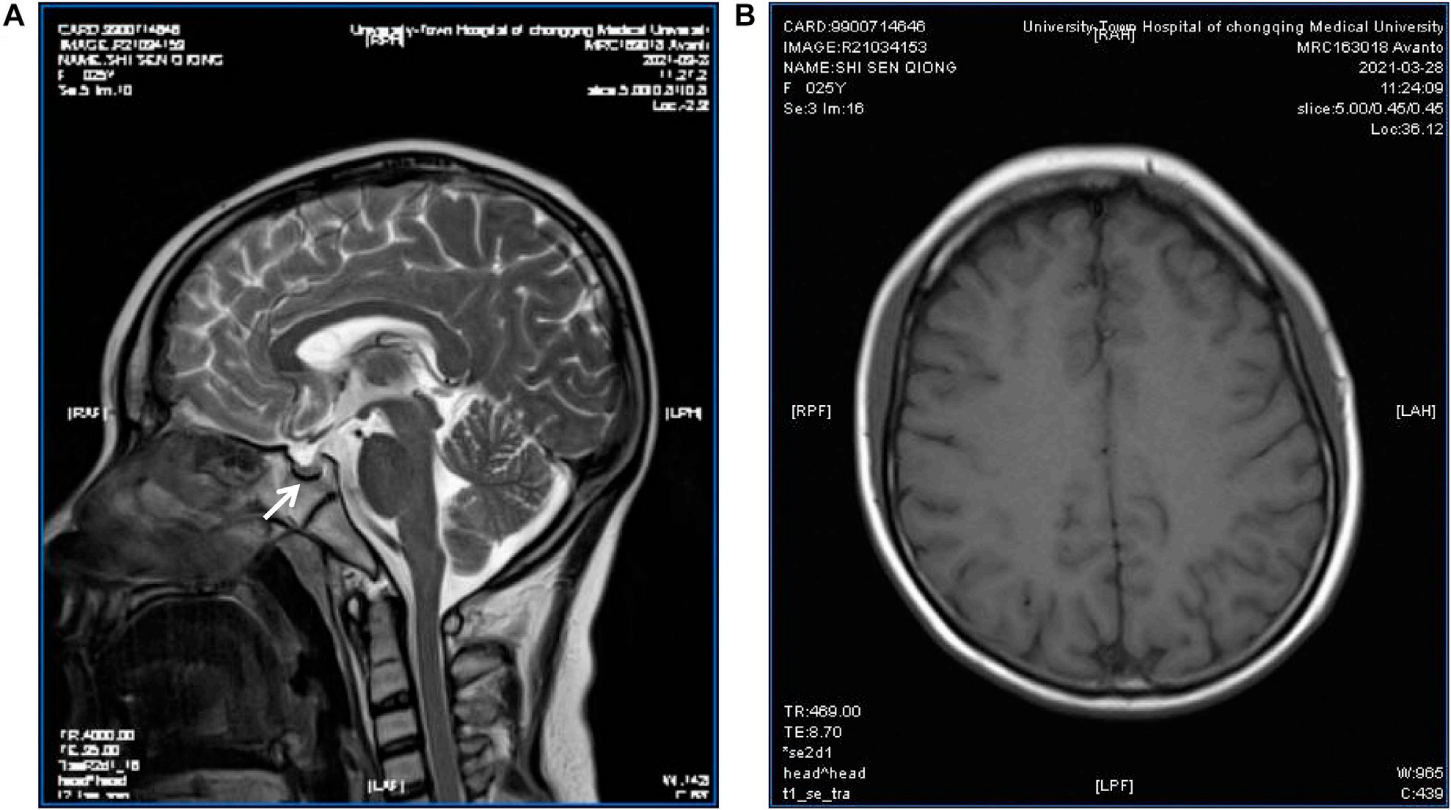
Pituitary MRI of the patient. T2-weighted MR imaging for the patient showed a partially empty sella and a small pituitary gland (arrow) **(A)**; Magnetic sensitivity weighted imaging showed no abnormal iron deposition accumulation **(B)**.

Sex hormone examinations disclosed low estradiol level (<5 pg/ml, reference range: 12.4–233 pg/ml) and low testosterone level (<0.025 pg/ml, reference range: 0.07–0.78 pg/ml). Her follicle-stimulating hormone level, prolactin level, and luteinizing hormone level were in the normal range. Other hormone investigations revealed high thyroid-stimulating hormone (6.36 uIU/ml, reference range: 0.27–4.2 uIU/ml), low free thyroxine (7.39 pmol/ml, reference range: 12–22 pmol/ml), and a normal free triiodothyronine level. These data were suggestive of subclinical hypothyroidism. She had a low insulin growth factor 1 (IGF-1) level (45.7 ng/ml, reference range: 69–200 ng/ml) and a high level of HbA1c (8.8%, reference range: 3.6–6%) (Table 1). Her C-peptide release test results were as follows: 0 min: 2.25 ng/ml, 30 min: 2.43 ng/ml, 60 min: 2.99 ng/ml, and 120 min: 5.58 ng/ml. A transabdominal ultrasound showed an immature uterus and small ovaries. Her breast ultrasound found tiny gland tissue in the bilateral breasts. A hearing assessment disclosed bilateral mild sensorineural deafness. A left wrist X-ray showed epiphyseal closure. An electrocardiograph (ECG) was unremarkable. Magnetic resonance imaging (MRI) revealed a small pituitary, partially empty sella, no iron deposits in the globus pallidus, and multiple intracranial white matter hyperintensities that may be of vascular origin (Figure 3). As mentioned above, the patient’s clinical features, metabolic disease, neurological findings, ectodermal appendages, and laboratory tests are summarized in Table 1.

**TABLE 1:** The patient’s main symptoms and laboratory tests.

Clinical features	
Sex	Female
Age(Yr)	25
Height (cm)	156
Weight (kg)	56
Hypoplastic secondary sexual characteristics	+
Few follicles	+
Prominent ears	+
No menstruation	+
Immature uterus	+
Small ovaries	+
**Metabolic disease**	
Diabetes mellitus	+
**Neurological findings**	
Normal MRI brain	+
Small pituitary	+
Intellectual disability	+
Dysaudia	+
Dysarthria	−
Dysphagia	−
Tremor	−
Abnormal muscle tension	−
Dyskinesia	−
**Ectodermal appendages**	
Alopecia	+
Delayed bone age	+
**Laboratory tests**	
Subclinical hypothyroidism	+
Sex hormone	<5 pg/ml
Testosterone	<0.025 pg/ml
Follicle-stimulating hormone	Normal
Prolactin	Normal
Luteinizing hormone	Normal
Free triiodothyronine	Normal
Thyroid-stimulating hormone	6.36 uIU/ml
Free thyroxine	7.39 pmol/ml
IGF-1	45.7 ng/ml
HbA1c	8.8%

+, presence of feature; −, absence of sign; IGF-1, insulin growth factor 1; Reference range of laboratory tests: Sex hormone: 12.4–233 pg/ml, Testosterone: 0.07–0.78 pg/ml, thyroid-stimulating hormone: 0.27–4.2 uIU/ml, free thyroxine: 12–22 pmol/ml, IGF-1: 69–200 ng/ml, HbA1c: 3.6–6%.

**FIGURE 4:**
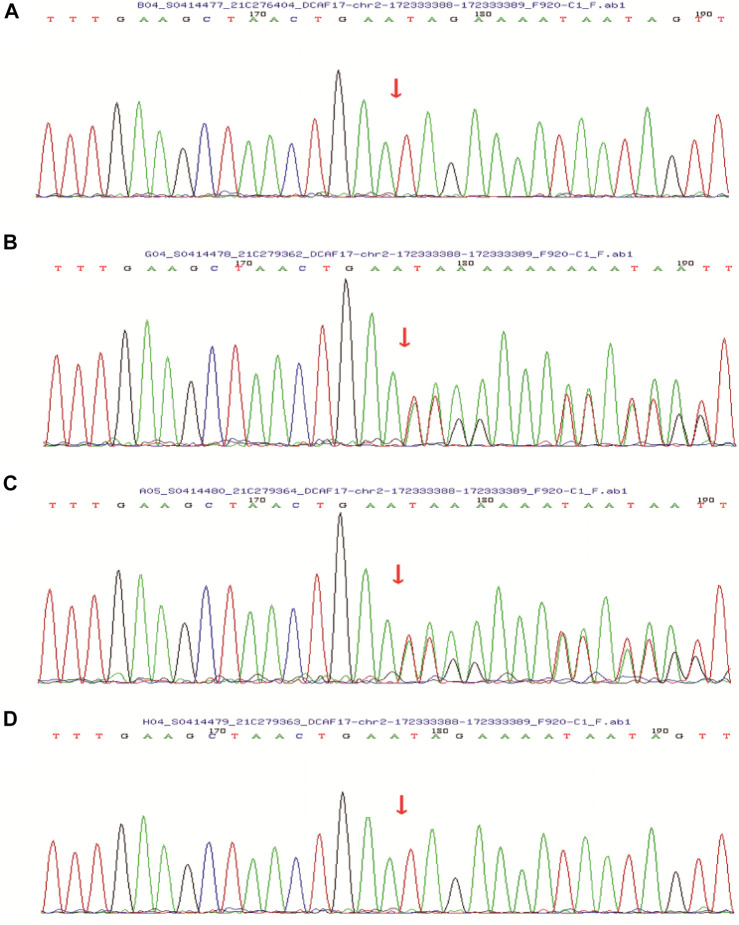
Sanger sequencing showed that the patient’s **(A)** and her younger brother’s **(D)** *DCAF17* harbored a homozygous mutation c.1111delA, p.(Ile371Term), while their father **(B)** and mother **(C)** were heterozygous for this mutation at this site.

After obtaining informed consent from the patient and her family, peripheral blood was sampled from the patient, her younger brother, and their parents. In order to better describe identification of the variant, we have added more detailed steps for genetic testing, data screening and pathogenicity analysis based on previous articles published after the company’s WES technical services (Ma et al., 2019; Zhang et al., 2019). We isolated nucleic acid to capture DNA fragments, then constructed a library, using biotinylated probes to target and capture exons, and amplify the captured targets, qPCR was used for quality control and sequencing, and to analyze the captured information. We analyzed DCAF17 mutatifons and their pathogenicity. We used the MGI-T7 platform (BGI) for sequencing at a depth of 200× and a target coverage of 99.57%. Forward primer: 3′-CAG​AAT​CTC​CGA​ATT​TGA​AGG​AG-5′, reverse primer: 3′-TCT​TTA​AAT​CTG​AAA​TGT​ACA​TGG​G-5’. Her parent’s and younger brother’s samples were tested *via* Sanger sequencing for verification (Supplementary Figure S1). With pathogenicity analysis (Zhang et al., 2019), we detected the pathogenic locus (Supplementary Figure S2). The patient’s *DCAF17* harbored a homozygous mutation c.1111delA, p.(Ile371Term), her younger brother had the same mutation, and her parents were heterozygous for the mutation at this site. The c.1111delA mutation is a nonsense mutation leading to a premature stop codon p.(Ile371Term) (Figure 4).

## Discussion

The most prominent manifestation of WSS is disorder of the neuroendocrine system (Agopiantz et al., 2014). Previously, reports revealed the presence of hypogonadism (100%), intellectual disability (87%), sensorineural hearing loss (76%), and extrapyramidal movements (42%) (Bohlega et al., 1993; Agopiantz et al., 2014). The first cases reported in 1983 were six Saudi Arabian patients from two highly inbred families (Woodhouse and Sakati, 1983). Until now, fewer than 100 WSS patients have been reported. The patient in this report sought medical treatment for 10 yr and was not diagnosed until she came to our hospital and we noticed that she had symptoms of WSS. This is consistent with previous reports (Schweitzer et al., 2003; Kurnaz et al., 2019; Louro et al., 2019; Shah et al., 2020), The patient presented with alopecia, hypogonadism, hearing loss, intellectual disability, and high blood sugar, but neither the patient nor her younger brother had other symptoms such as dysarthria, dyskinesia with oropharyngeal dysphagia, or difficulty walking, and no abnormal manifestations such as iron deposition on the MRI image. In this case, the mutation site was different from previous reports. Meanwhile, this is the first reported Chinese WSS family.

The *DCAF17* gene, located on chromosome 2q31.1, encodes a nucleolar protein with two isoforms including alpha (453 amino acid residues; NP_001158293.1) and beta (520 amino acids residues; NP_079276.2) (Bohlega et al., 1993; Andersen et al., 2005). The protein is expressed in the brain, liver, and skin, as well as in seminiferous tubules in male mice, a pattern that is compatible with the neurosensory, endocrine, and cutaneous phenotypic manifestations (Agopiantz et al., 2014). Previous literature regarding this nucleolar protein showed that it functions as a substrate co-receptor for the ubiquitin ligase complex Cul4-DDB1 (Angers et al., 2006; Ali et al., 2016), which emphasizes the lesser-known functions of the nucleolus as a caretaker of normal development and post-development maintenance, including the cellular activities of growth, proliferation, and response to stress as well as in the cell cycle and apoptosis (Lee and Zhou, 2007). Mutation of *DCAF17* leads to disruption of the nucleolus, and this damage results in dysregulated ribosome biogenesis, regulation of the cell cycle, cellular aging, signal recognition, small-RNA processing, mRNA transport, and apoptosis (Andersen et al., 2005). These disruptions may underlie the pathogenesis of WSS. Nevertheless, the pathogenic mechanism underlying the clinical variability associated with different mutations has not been explored extensively. Further experimental studies are necessary to reveal the exact pathogenic mechanisms of how these genes affect different tissues.

The mutation c.1111delA, p.(Ile371Term) in our case resulted in an adenine deletion at basepair 1111, and this deletion leads to translation termination as stated in the literature (Ali et al., 2016), We speculate that an incomplete protein with missing domains and motifs necessary to interact with the DDB1-CUL4 ubiquitin ligase complex and recruit substrates was formed. Another possible result of the mutation would be degeneration of gene transcript through mRNA decay (Kurosaki et al., 2021; Yang et al., 2021). This nonsense mutation was different from other nonsense mutations in previous reports (Bohlega et al., 1993; Boisvert et al., 2007; Alazami et al., 2010; Habib et al., 2011; Sheridan et al., 2015).

In conclusion, WSS is a genetic disorder of the neuroendocrine system that leads to neuroendocrine cell aging function loss, which is usually caused by mutations in the *DCAF17* gene. Significantly, we have identified a novel mutation in the *DCAF17* gene. Molecular genetic analyses should be performed as a crucial tool to inform early diagnosis. Additional work to explore the clinical and underlying biological mechanisms will be helpful to further understand the disease.

## Data Availability

The datasets presented in this study can be found in online repositories. The names of the repository/repositories and accession number(s) can be found in the article/Supplementary Material.
