# Supplementary material for: Case Report: A Deletion Variant in the DCAF17 Gene Underlying Woodhouse-Sakati Syndrome in a Chinese Consanguineous Family
Source: Front Genet. 2021 Sep 23;12:741323. doi: 10.3389/fgene.2021.741323 (PMC8498701; doi:10.3389/fgene.2021.741323)
Supplement: Supplementary file 1 [file DataSheet1.docx]

Supplementary Table 1. The mutation details of *DCAF17* leading to WSS.

| **Gene location** | **Reference**  **base** | **Mutant base** | **Mutation information** | **HGVS** | **Type of mutation** |
| --- | --- | --- | --- | --- | --- |
| 2:172305210-172305210 | C | A | Ser114Term | 341C>A | Nonsense mutation |
| 2:172325465-172325465 | G | A | Trp302Term | 906G>A |  |
| 2:172305256-172305256 | G | A | Trp129Term | 387G>A |  |
| 2:172330486-172330486 | G | A | IVS10 ds G-A +1 | 1091+1G>A | Intronic mutation |
| 2:172300124-172300124 | G | A | IVS3 ds G-A +1 | 321+1G>A |  |
| 2:172330487-172330487 | T | C | IVS10 ds T-C +2 | 1091+2T>C |  |
| 2:172336708-172336708 | G | T | IVS13 ds G-T +5 | 1422+5G>T |  |
| 2:172330491-172330491 | T | G | IVS10 ds T-G +6 | 1091+6T>G |  |
| 2:172330388-172330389 | AT | A | del 1 bp codon 332 | 995delT | Frameshift mutation |
| 2:172334550-172334551 | TA | T | del 1 bp codon 413 | 1238delA |  |
| 2:172300068-172300068 | C | CA | ins 1 bp codon 91 | 270dupA |  |
| 2:172291589-172291592 | TTAG | TAA | del 3 bp / ins 2 bp non-coding DNA | 127-3_127-1delTAGinsAA |  |
| 2:172300068-172300069 | CA | C | del 1 bp codon 90 | 270delA |  |
| 2:172300085-172300085 | G | GA | ins 1 bp codon 97 | 289dupA |  |
| 2:172305304-172305305 | TC | T | del 1 bp codon 146 | 436delC |  |
| 2:172306381-172306429 | TTTAAAAGATACTTGAGCTGGGACACTCCTCAAGAAGTCATTGCAGTTA | T | deletion 48 bp, c.459-7_499 | 459-7_499del48 |  |
| 2:172291136-172291137 | GC | G | del 1 bp codon 17 | 50delC |  |
| 2:172291088-172291088 | A | G | Met1Val | 1A>G | Start loss mutation |


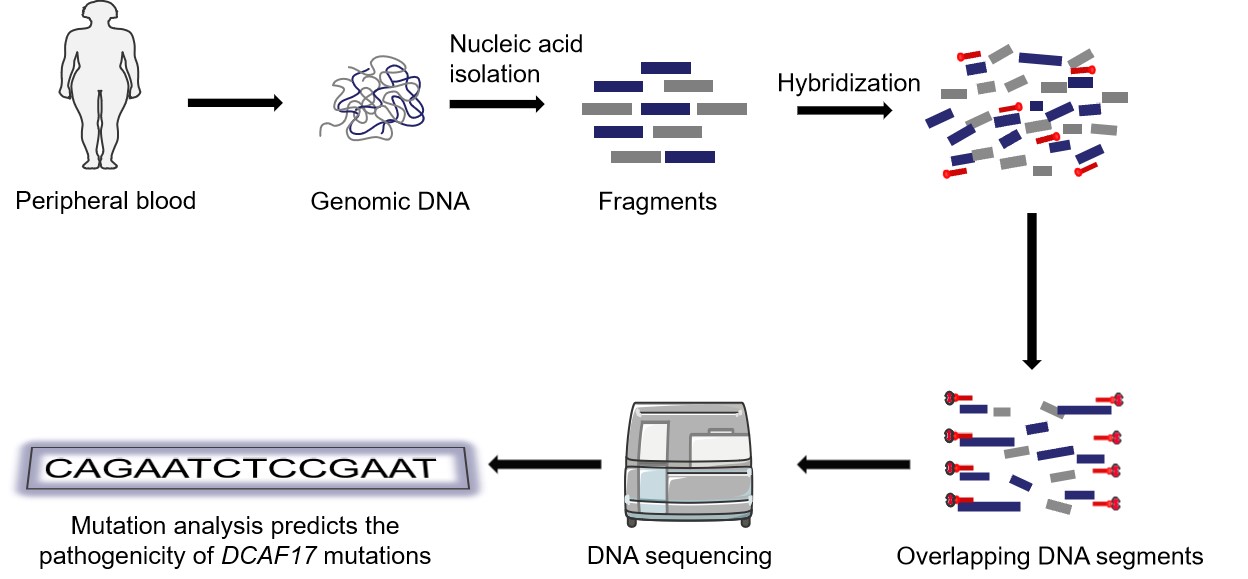
Supplementary Figure 1. Principle diagram of WES gene detection.


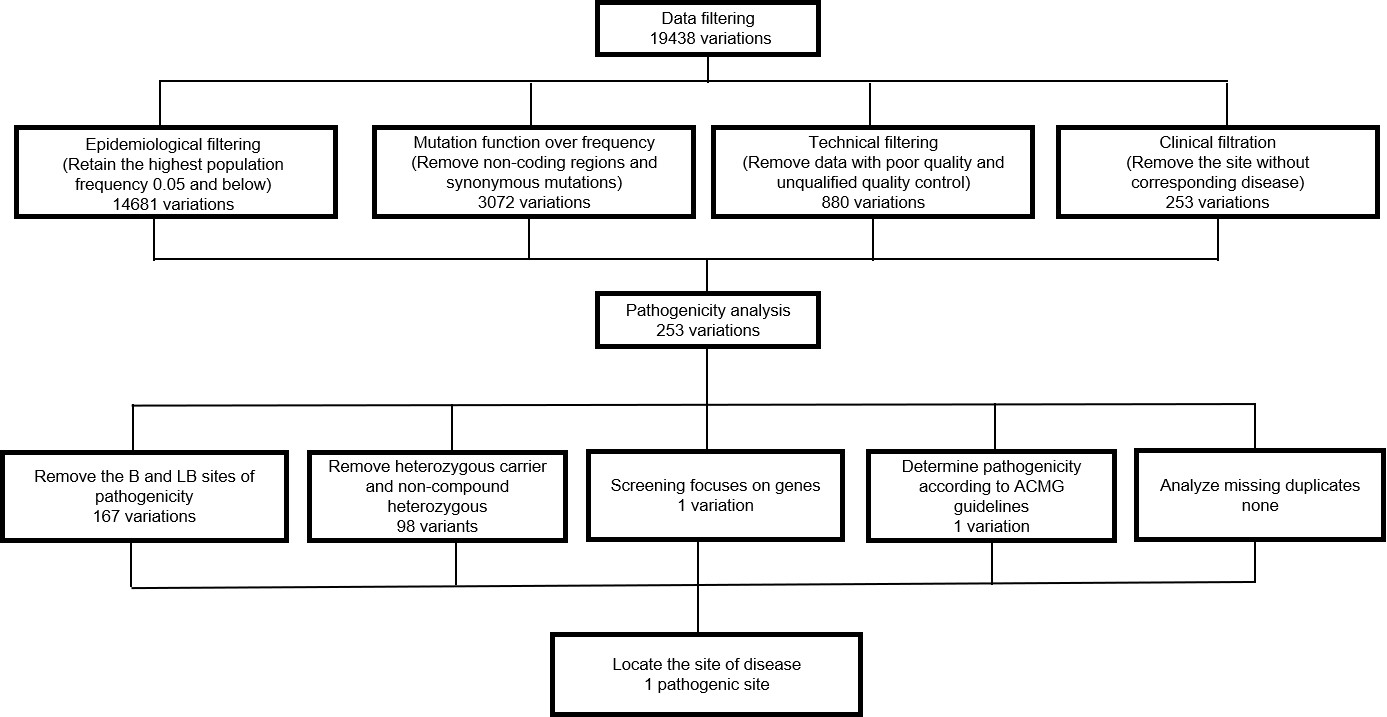


Supplementary Figure 2. Workflow diagram of genetic analysis.
